# Genome-wide comparative analyses of correlated and uncorrelated phenotypes identify major pleiotropic variants in dairy cattle

**DOI:** 10.1038/s41598-017-09788-9

**Published:** 2017-08-23

**Authors:** Ruidong Xiang, Iona M. MacLeod, Sunduimijid Bolormaa, Michael E. Goddard

**Affiliations:** 1 Faculty of Veterinary & Agricultural Science, University of Melbourne, Parkville, Victoria, 3010 Australia; 2 AgriBio, Department Economic Development, Jobs, Transport & Resources, Bundoora, Victoria, 3083 Australia; 3 Cooperative Research Centre for Sheep Industry Innovation, Armidale, NSW 2351 Australia

## Abstract

While single nucleotide polymorphisms (SNPs) associated with multiple phenotype have been reported, the knowledge of pleiotropy of uncorrelated phenotype is minimal. Principal components (PCs) and uncorrelated Cholesky transformed traits (CT) were constructed using 25 raw traits (RTs) of 2841 dairy bulls. Multi-trait meta-analyses of single-trait genome-wide association studies for RT, PC and CT in bulls were validated in 6821 cows. Most PCs and CTs had substantial estimates of heritability, suggesting that genes affect phenotype via diverse pathways. Phenotypic orthogonalizations did not eliminate pleiotropy: the meta-analysis achieved an agreement of significant pleiotropic SNPs (*p* < 1 × 10^−5^, n = 368) between RTs (416), PCs (466) and CTs (425). From this overlap we identified 21 lead SNPs with 100% validation rate containing two clusters: one consisted of *DGAT1* (chr14:1.8 M+), *MGST1* (chr5:93 M+), *PAEP* (chr11:103 M+) and *GPAT4* (chr27:36 M+) affecting protein, milk and fat yield and the other included *CSN2* (chr6:87 M+), *MUC1* (chr3:15.6 M), *GHR* (chr20:31.2 M+) and *SDC2* (chr14:70 M+) affecting protein and milk yield. Combining beef cattle data identified correlated SNPs representing *CAPN1* (chr29:44 M+) and *CAST* (chr 7:96 M+) loci affecting beef tenderness, showing pleiotropic effects in dairy cattle. Our findings show that SNPs with a large effect on one trait are likely to have small effects on other uncorrelated traits.

## Introduction

Understanding genetic control of mammalian phenotype, including body growth, health outcomes and metabolic pathways can improve patient treatment^1^, knowledge of evolution^2^ and agricultural efficiency^3^. Most mammalian phenotypes are quantitative or complex traits, whose variation is controlled by many genomic mutations with small effects and by environmental effects. While thousands of single nucleotide polymorphisms (SNPs) have been found associated with individual complex traits by genome-wide association studies (GWAS), an important question is the extent to which the same causal variants affect multiple traits, i.e. the extent of pleiotropy^4^.

It is expected that correlated traits share some causal variants and this has been observed in humans^5^ and livestock^6, 7^. However, it is also possible that uncorrelated traits share some causal variants. This possibility can be tested by transforming a set of correlated traits into uncorrelated traits, for instance, by principal components (PCs) analysis^8, 9^. If genes influence a set of traits through a limited number of physiological pathways, it may result in that only the first few PCs showed strong genetic effects leading to a simple picture of pleiotropy. A previous study analysing simulated and a small amount of real human data showed that large genetic variances can exist in PCs explaining small amount of total phenotypic variances^10^.

To further describe the extent and nature of pleiotropy in large mammals, we used data on 2841 progeny-tested dairy bulls with phenotypes on 25 traits and high density genotypes of 632,002 SNPs. Whilst many pleiotropic SNPs have been found in beef cattle^6^, an equivalent analysis of a large number of dairy traits has not been conducted, although some pleiotropic patterns were reported^9^. The 25 dairy traits included measures of milk production, fertility^11^, conformation and management traits^12^ which contribute to the profitability of dairy farming^13^. Secondly, we examined the effects in dairy cattle of SNPs significantly associated with quantitative traits in beef cattle^7^.

We used genome-wide meta-analysis modelling Chi-square distributions of SNP effect size^7^ to analyse 25 raw traits (RTs), 25 PCs and a novel set of phenotypic orthogonalisation, Cholesky transformed traits (CTs)^14^ in the dairy cattle discovery population. This was followed by the selection of lead SNPs representing major dairy QTL, confirmed by conditional and joint analysis. Finally, SNPs associated with both dairy traits alone and those SNPs shared by dairy and beef traits were validated at both multi-trait (linear index approach) and single-trait (GWAS) level in a separate population of 6821 dairy cows.

## Methods

### Animals, genotypes and phenotypes

No live animals were used in this study. Phenotype data (trait deviations for cows and daughter trait deviations for bulls, Table 1) were from the April 2016 genetic evaluations from DataGene (http://www.datagene.com.au/). Daughter trait deviations were the average trait deviations of a bull’s daughters and all phenotypes were pre-corrected for known fixed effects. Only those bulls’ phenotype which were based on records from more than 15 progenies were included. Complete phenotype data were from 9,662 dairy cattle from the breeds Holstein, Jersey, MIX (crosses between Holstein and Jersey) and Australian Red, a genetically distinct breed^15^ (Supplementary Table S1). All animals had either real or imputed high density array genotype data following previous procedures^15^ and in total, 632,002 SNPs were used after quality control^15, 16^. SNPs with minor allele frequency <0.01 or departing from Hardy-Weinberg equilibrium (p < 0.001) were discarded. 2,841 bulls were used as the discovery population due to the high accuracy of the phenotype which was the average of >15 daughter records. The 6,821 cows, who had individual phenotypic records and weak genetic relationships with the bulls (Supplementary Figure S1), were used as the validation population.

**Table 1:** Summary of single-trait genome-wide studies (GWAS) results for raw traits, principal components (PCs) and Choleskey transformed traits (CTs).

Raw traits	Full names	trait type	^1^SNPs	h^2^	se	PC	Eigenvalues	Vp^2^	^1^SNPs	h^2^	se	CT	^1^SNPs	h^2^	se
01.Prot	protein yield	production	119	0.86	0.02	PC1	6.355	0.254	5	0.67	0.03	01.Prot	119	0.86	0.02
02.Fat	fat yield	production	178	0.82	0.02	PC2	2.504	0.100	3	0.61	0.03	02.Fat	408	0.85	0.02
03.Milk	milk yield	production	230	0.86	0.02	PC3	2.123	0.085	21	0.53	0.03	03.Milk	344	0.90	0.01
04.SCC	somatic cell count	production	8	0.82	0.02	PC4	1.758	0.070	26	0.65	0.03	04.SCC	8	0.82	0.02
05.SurvDi	survival	reproduction	27	0.53	0.03	PC5	1.459	0.058	3	0.55	0.03	05.SurvDi	14	0.45	0.03
06.Fert	fertility	reproduction	31	0.54	0.03	PC6	1.145	0.046	25	0.56	0.04	06.Fert	6	0.43	0.03
07.Temp	temperament	management	6	0.50	0.03	PC7	1.004	0.040	9	0.49	0.03	07.Temp	49	0.47	0.03
08.MSpeed	milking speed	management	13	0.57	0.03	PC8	0.910	0.036	28	0.57	0.03	08.MSpeed	10	0.54	0.03
09.Stat	stature	linear type	19	0.64	0.03	PC9	0.881	0.035	6	0.57	0.04	09.Stat	25	0.63	0.03
10.Like	likeability	management	4	0.48	0.03	PC10	0.809	0.032	44	0.57	0.03	10.Like	1	0.16	0.03
11.Angul	angularity	linear type	1	0.37	0.04	PC11	0.741	0.030	14	0.43	0.04	11.Angul	1	0.24	0.03
11.Bone	bone quality	linear type	6	0.49	0.03	PC12	0.718	0.029	8	0.52	0.03	11.Bone	5	0.41	0.04
11.CentL	central ligament	linear type	5	0.47	0.03	PC13	0.611	0.024	43	0.41	0.04	11.CentL	6	0.36	0.04
11.ChestW	chest width	linear type	9	0.49	0.03	PC14	0.584	0.023	1	0.52	0.03	11.ChestW	15	0.36	0.03
11.ForeA	fore attachment	linear type	3	0.47	0.04	PC15	0.486	0.019	42	0.44	0.04	11.ForeA	2	0.36	0.04
11.MuzW	muzzle width	linear type	16	0.43	0.03	PC16	0.444	0.018	6	0.38	0.03	11.MuzW	7	0.33	0.03
11.PinSet	pin set	linear type	12	0.62	0.03	PC17	0.437	0.017	42	0.37	0.03	11.PinSet	14	0.58	0.04
11.PinW	pin width	linear type	9	0.56	0.03	PC18	0.416	0.017	241	0.53	0.03	11.PinW	5	0.48	0.03
11.RSet	rear legs set	linear type	15	0.35	0.04	PC19	0.356	0.014	129	0.31	0.03	11.RSet	4	0.33	0.03
11.RearAH	rear attachment height	linear type	12	0.62	0.03	PC20	0.330	0.013	3	0.22	0.03	11.RearAH	4	0.47	0.04
11.RearAW	rear attachment width	linear type	12	0.51	0.03	PC21	0.272	0.011	6	0.27	0.03	11.RearAW	1	0.37	0.04
11.TeatPF	front teat placement	linear type	1	0.69	0.03	PC22	0.252	0.010	2	0.09	0.03	11.TeatPF	4	0.57	0.03
11.UdTex	udder texture	linear type	4	0.41	0.03	PC23	0.195	0.008	3	0.12	0.03	11.UdTex	0	0.08	0.03
24.OType	overall type	linear type	5	0.49	0.03	PC24	0.140	0.006	44	0.35	0.03	24.OType	5	0.16	0.03
25.Mamm	mammary system	linear type	7	0.50	0.03	PC25	0.070	0.003	153	0.77	0.02	25.Mamm	13	0.31	0.03

^1^The number of SNPs with single-trait GWAS *P* < 1 × 10^−5^. ^2^Total phenotypic variances explained by each principal component.

### Phenotype orthogonalisation

The principal components (PCs) and Cholesky transformed traits (CTs) were calculated based on centered and z-score scaled raw traits (RTs) of the discovery population in R (v3.3.1)^17^. Given *n* number of animals and *k* number of RTs, an *n* × *k* matrix of PC scores was calculated based on eigen-decomposition:

1 $${u}_{n}=T^{\prime} {g}_{n}$$

where *u*_*n*_ was a *k* × 1 vector of PC scores for animal *n*; *T* was a *k* × *k* matrix of eigenvectors such that the variance matrix of the PC Var(T’g) = D, a diagonal matrix of eigenvalues; *g*_*n*_ was an k × 1 vector of RT for animal *n*. The 25 eigenvectors were shown in Supplementary Table S2. The *n* × *k* CT matrix was calculated based on Cholesky decomposition:

2 $${c}_{n}={L}^{-1}{g}_{n}$$

where *c*_*n*_ was a *k* × 1 vector of Cholesky scores for the animal *n*; *L* was the *k* × *k* matrix of the Cholesky factor which satisfied *LL* ^*t*^ = *COV*, the *k* × *k* covariance matrix of raw scores after standardisation as z-scores^14^; *g*_*n*_ was an *k* × 1 vector of RT for animal *n*. The *k*th CT can be interpreted as the *k*th raw trait corrected for the preceding *k*-1 traits. Consequently, 1^st^ CT equals the 1^st^ RT.

### Single-trait genome-wide association studies(GWAS) in the discovery population

25 RTs with the derived sets of 25 PCs and 25 CTs were analysed one trait at a time with linear mixed models using GEMMA^18^:

3 $$y=mean+bree{d}_{i}+bx+a+error$$

where y = vector of phenotypes for bulls (discovery population), *breed*_i_ = three breeds, Holstein, Jersey and Australian Red; *bx* = regression coefficient *b* on SNP genotypes *x*; *a* = polygenic random effects ~N(0, Gσ_g_^2^) where G = genomic relatedness matrix based on all SNPs^18, 19^. The same model was applied to GWAS and, without including SNP in the model, calculations of SNP heritability for all RTs, PCs and CTs.

The count of SNPs that were significant (single-trait GWAS *p*_s_ < 0.05) for both of a pair of traits were compared with the expected number using the Fisher’s exact test (*p*_f_) implemented in GeneOverlap^20^ in R.

### Multi-trait meta-analysis

Multi-trait meta-analysis of 25 RTs or 25 PCs or 25 CTs followed previous procedures^6, 7^. Briefly, the multi-trait χ^2^ statistic for the *i*th SNP was calculated based on its signed t values generated from each single trait GWA:

4 $${\chi }^{2}=t{^{\prime} }_{i}{V}^{-1}{t}_{i}$$

where *t*_*i*_ was a *k* (number of traits = 25) × 1 vector of the signed t-values of SNP_*i*_ effects, i.e., beta/se, for the *k* traits; *t*_*i*_′ was a transpose of vector *t*_*i*_ (1 × *k*); and V^−1^ was an inverse of the *k* × *k* correlation matrix where the correlation was calculated over the all estimated SNP effects (signed t-values) of the two traits. The χ^2^ value of each SNP was examined for significance based on a χ^2^ distribution with *k* degrees of freedom to test against the null hypothesis that the SNP had no significant effects on any one of the *k* traits. The false discovery rates of χ^2^ tests were calculated following Storey’s method^21^ by ‘qvalue’ package in R. An additional test of pleiotropy was performed by carrying out the multi-trait test separately for odd and even numbered PCs. This was to confirm the existence of pleiotropy using PCs’ orthogonality: if a SNP had strong pleiotropic effects on all PCs, the variances of which were randomly attributable to RTs, this SNP was then expected to have strong effects on any subsets of PCs. Following this logic, this pleiotropic SNP was expected to have consistently strong effects on randomly selected odd and even PCs. t values of single-trait GWA of odd PCs and of even PCs were combined to calculated odd PC χ^2^ and even PC χ^2^. We then tested whether or not the same SNPs were significant in both analyses.

### Selection of dairy cattle lead SNPs

The selection of lead SNPs representing major QTLs was based on the most significant SNP (multi-trait meta-analysis *p*_m_ of RT, PC and CT at least <1 × 10^−5^) within non-overlapping 1-Mb intervals of a chromosome of the discovery population. For chromosomes with multiple significant SNPs, the 1-Mb interval started from the most significant SNP and approached to the distal ends. Such selected lead SNPs were refined by step-wise analyses (similar to ref. 19): firstly, the lead SNPs were fitted as covariates simultaneously for each single-trait GWAS of RT, PC and CT. t values of such single-trait GWAS were used for meta-analysis described above to determine multi-trait significance. While most SNPs, after adjusting for the existing lead SNPs’ effects, were insignificant, a few SNPs were still significant (*p*_m_ of RT, PC and CT all <1 × 10^−5^). The most significant ones of these few SNPs one per 1 Mb interval were again selected, added to the list of existing lead SNPs and the process repeated until there were no additional SNPs significant after fitting the lead SNPs. Then, joint analyses^22^ fitting all lead SNPs in a regression was used to estimate their effects on traits. Those SNPs without significant effects (joint P < 0.05) on at least one trait were removed from the lead SNP list.

### Cluster analysis

Those lead SNP effects (above generated t values) that had the same direction of effect in both the discovery and validation (see below) were used to calculate the effect correlation matrix within RTs, PCs and CTs. The correlation matrices were used to perform hierarchical clustering.

### SNP annotation

Genes associated with SNPs were annotated by variant effect predictor^23^ and previous publications including^7, 15, 24–27^. Published results overrode ensemble predicted annotation if both existed. If no genes from Ensembl or published results could be identified for the SNP, the closest gene within 1 Mb was assigned.

### Selection of dairy and beef cattle shared SNPs

Beef cattle traits with animal numbers >1900 from a previous study (Table 1 in ref. 7) were selected. t values of these traits were used for meta-analysis as described above. Beef cattle SNPs with *p*_m_ < 1 × 10^−5^ in the meta-analysis were selected and compared with dairy cattle SNPs with *p*_m_ < 0.05 in the meta-analysis of 25 dairy RTs. The significance of the overlap between SNPs significant in the dairy and beef analysis was tested with the GeneOverlap.

### Validation using cow data

The phenotypes on the validation population (i.e. the cows) did not include one trait (‘Mamm’), so the multi-trait analysis on the bulls was repeated with 24 traits so that the results could be compared directly with results on the cows. Based on these results the linear index of 24 traits on which each lead SNP had the most significant effect was calculated by:

5 $${y}_{i}=\{\begin{array}{c}{b}_{RT}^{\prime} {{C}^{-1}}_{RT}{y}_{RT}\\ {b}_{PC}^{\prime} {{C}^{-1}}_{PC}{y}_{PC}\\ {b}_{CT}^{\prime} {{C}^{-1}}_{CT}{y}_{CT}\end{array}$$

*b*′ was the transpose of a vector of the size effects (beta) of the SNP (to be validated) on the 24 RT, PC and CTs; *C* ^−1^ was an inverse of the 24 × 24 (co)variance matrix among the *k* traits calculated from the beta of all tested SNPs for RT, PC and CTs only in the discovery population. y_RT_ was a 24 × 1 vector of the phenotype values for the 24 traits (matching 24 RTs in bulls) for each animal in the validation population. y_PC_ and y_CT_ in the validation population were calculated using the same formula for calculating 24 PCs (PC eigenvectors) and 24 CTs (Cholesky L matrix) as in the discovery population according to ref. 9. The linear index (*y*_*i*_) for each cow in the validation population was analysed as a new response variable for an association with only the lead SNP_*i*_ used to define this linear index. Such linear index analysis was also applied to validate in dairy cows the SNPs that were significant in the analysis of beef cattle and the dairy bulls. Dairy lead SNPs and dairy-beef shared SNPs were also validated for individual RT by comparing SNP effect directions between GWAS of the discovery and validation populations.

### Data availability

The genotype data used in this study were included in published articles^15^ and ref. 7. Supporting data were included in Supplementary Tables S1–7.

## Results

### Single-trait GWAS of RTs, PCs and CTs

Among the RTs and CTs there was a tendency for the traits with the highest heritability to have the highest number of significant SNPs (Table 1). Milk production, i.e., protein (01.Prot), fat (02.Fat) and milk (03.Milk) yield RTs and CTs had the largest numbers of significant SNPs (>100) and had the highest estimated heritability (>0.8). Reproduction and behaviour traits including survival and fertility, temperament and milking speed had mid-range heritability estimates and reduced number of significant SNPs compared to milk production traits. Conformation or type traits had mid to low range heritability estimates and a small number of significant SNPs (<20).

The single trait analysis of the PCs’ showed little relationship between the phenotypic variance explained by each PC, the number of significant SNPs and the heritability (Table 1). PC1 (which had moderate loadings from many traits, Supplementary Table S2) accounted for 25% of the total variance across all traits, but had only 5 significant SNPs and a moderate estimation of heritability (0.67). Conversely, PC25 (high positive loading for protein yield and high negative loading for milk yield) explained 0.03% of the variances in all traits, yet also had a modest estimation of heritability (0.50 ± 0.03) and l53 significant SNPs.

### Shared SNPs at the single-trait GWAS level

**Figure 1:**
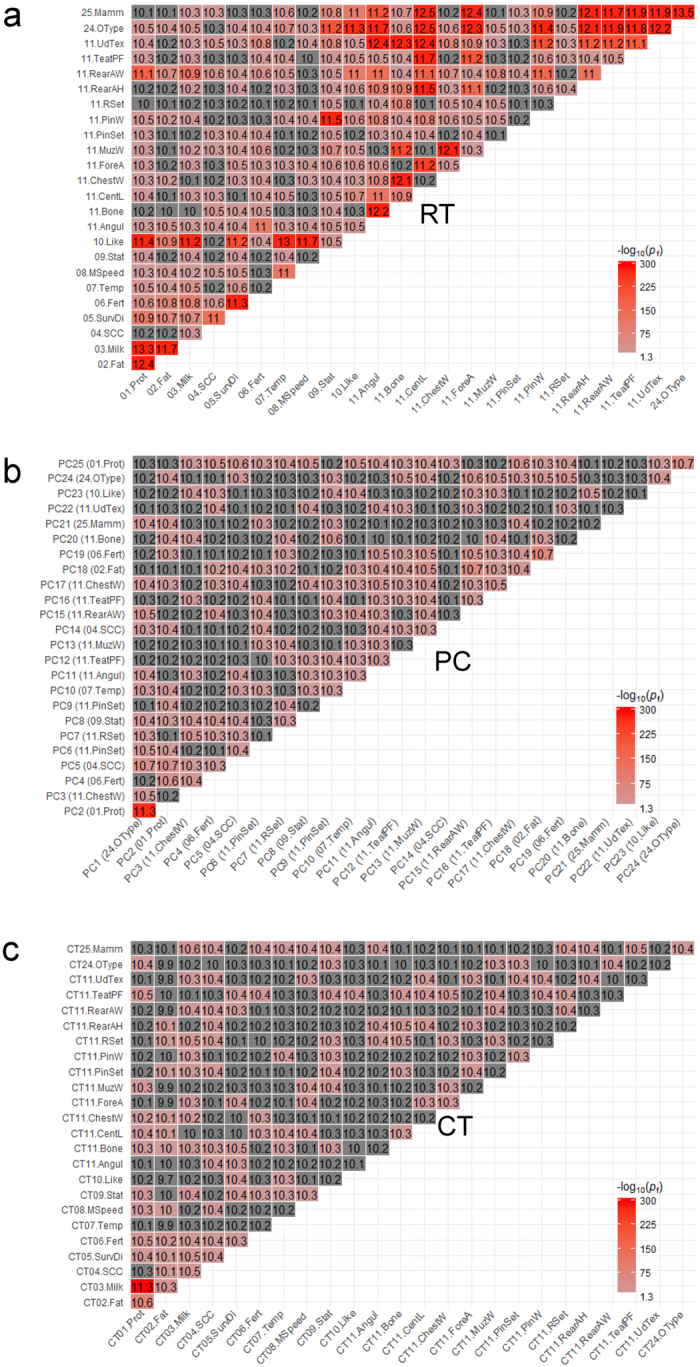
Overlap of SNPs detected by single-trait GWAS (*p*_s_ < 0.05) within raw traits (RTs, **a**), principal components (PCs, **b**) and Cholesky transformed traits (CTs, **c**). Numbers in cells were rounded log2 count of shared SNPs between single-trait GWAS pairs. Overlap significances were based on Fisher’s exact tests (*p*_f_) accounting for the number of SNPs of each pair of single-trait GWAS and the total number of SNPs analysed. The RT associated with the top factor loading value of each PC was shown in the parentheses (Supplementary Table S2).

Of 576 pairs of RT, 494 pairs had more SNPs with significant (*p*_s_ < 0.05) effects on both traits than expected by chance (Fisher’ exact test, *p*_f_ < 0.05) (Fig. 1a). Although the number of pairs of traits sharing significant SNPs was reduced among the uncorrelated traits, PC (Fig. 1b) and CTs (Fig. 1c) still had 423 and 394 out of 576 pairs, respectively with more shared SNPs than expected by chance. Milk production related traits, e.g., RT protein yield, PC2 (top loading associated with protein yield) and CT milk yield tended to have a large number of significant SNPs that were also significant for other traits (Fig. 1).

**Figure 2:**
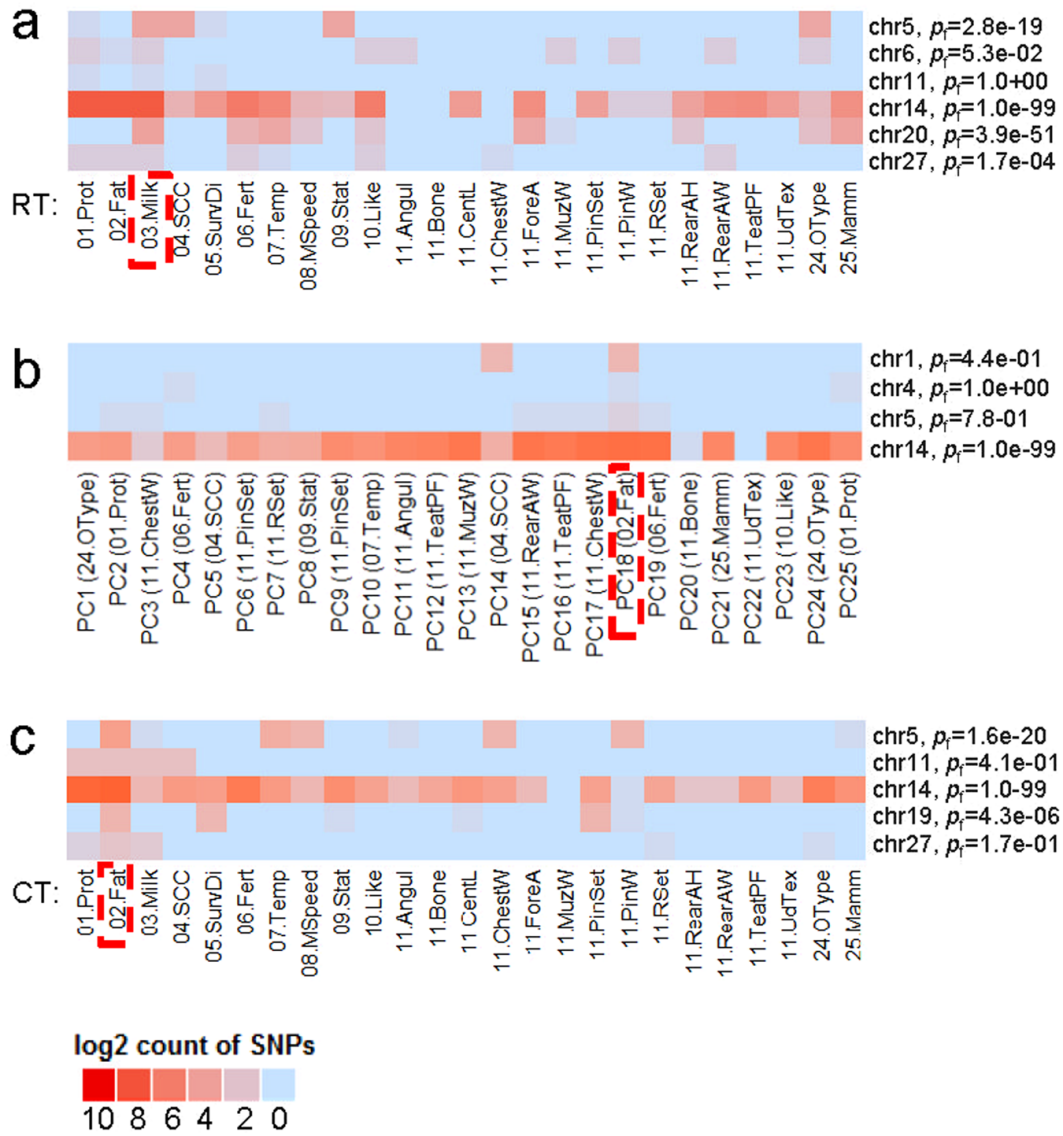
The breakdown of shared significant SNPs detected for selected traits with the other traits on each chromosome. As highlighted in red dashed boxes, selected milk yield raw trait (**a**), principal component 18 (**b**) and Cholesky transformed fat yield (**c**) had the largest numbers of significant (*p*_s_) SNPs detected by single-trait GWAS for RT, PC and CT, respectively (Table 1). The number of shared SNPs for the each other trait (non-selected) were determined at the *p*_s_ < 0.05 level (see methods). Significances of the enrichment of chromosomes containing the amount of significant SNPs detected for selected traits were based on the Fisher’s exact test (*p*_f_). The RT associated with the top factor loading value of each PC was shown in the parentheses (Supplementary Table S2).

Among the single trait GWAS of the RTs, milk yield had the largest number of significant SNPs (Table 1) and shared many significant SNPs with other traits (Fig. 1a). Figure 2a showed that these shared SNPs were concentrated on chromosome 14 and, to a lesser extent, on chromosomes 5, 6, 20 and 27. Figure 2b gave a similar breakdown of SNP shared by PC18 and Fig. 2c showed the same for CT fat. Chromosome 14, containing the *DGAT1* locus which is strongly associated with milk and fat traits^28^, also had the largest number of significant SNPs segregating across single-trait GWAS for RTs, PCs and CTs (Fig. 2a–c). Chromosome 5, containing the *MGST1* locus associated with milk fat percentages^25^, had significant enrichment of SNPs affecting milk production related RTs and CTs (Fig. 2a,c). SNPs affecting RT milk were also significantly enriched for chromosome 20 and 27, containing milk yield loci *GHR* ^15^ and fat percentage loci *GPAT4* ^29^, respectively.

### Multi-trait meta-analysis to detect pleiotropy in RTs, PCs and CTs

**Figure 3:**
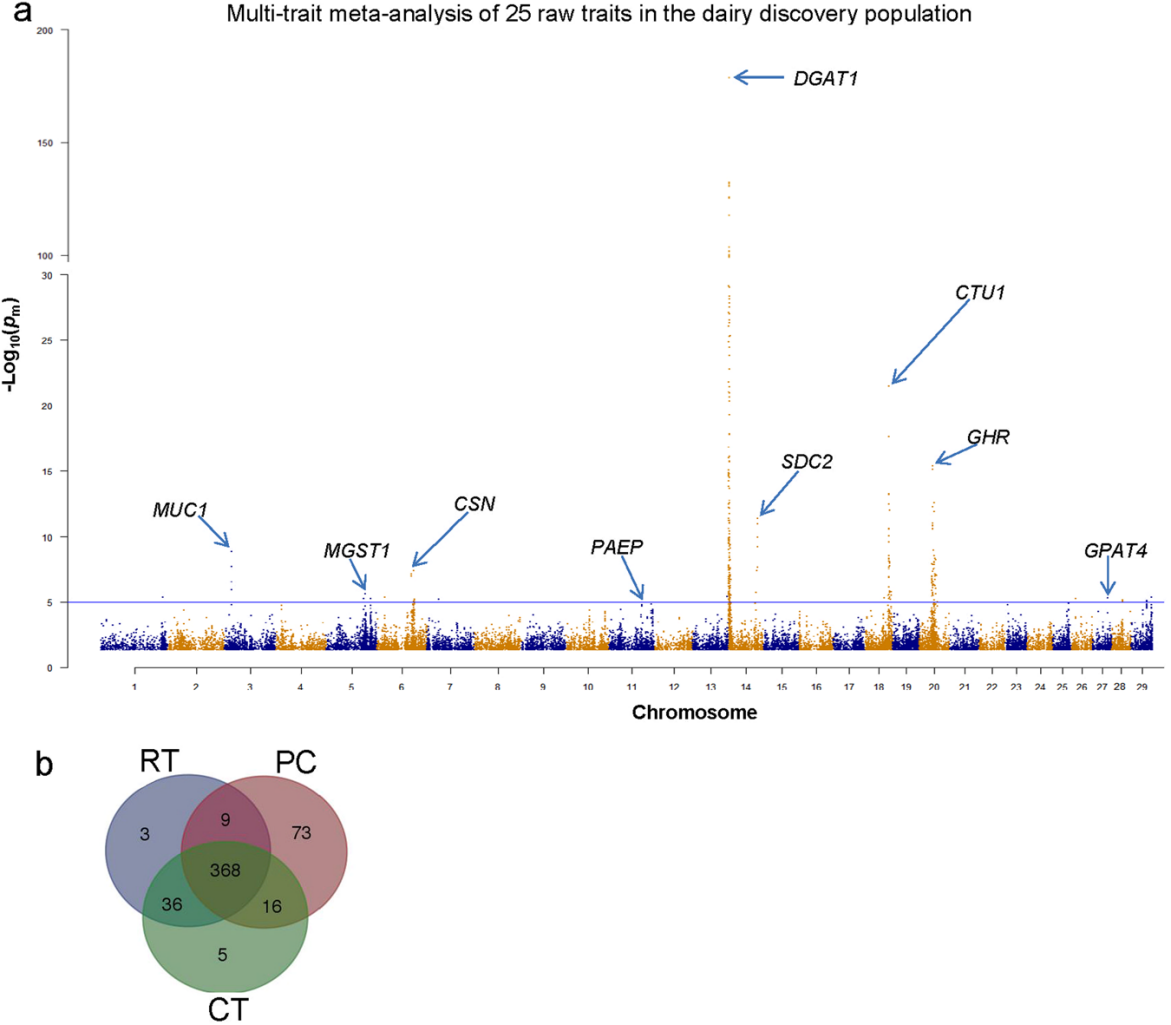
Summary of multi-trait meta-analysis of 25 raw traits (RTs) in the discovery population (dairy bulls). (**a**) Manhattan plot using SNPs with multi-trait meta-analysis *p*_m_ < 0.05. The horizontal blue line was *p*_m_<= 1 × 10^−5^. Some reported loci affecting milk traits were highlighted. Equivalent Manhattan plots of principal components (PCs) and Cholesky transformed traits (CTs) were shown in Supplementary Figure S3. (**b**) Venn gram showing the overlap of numbers of significant (*p*_m_ < 1 × 10^−5^) SNPs from multi-trait meta-analysis of RTs, PCs and CTs in the discovery population.

Three multi-trait analyses were performed. The number of significant (*p*_m_ < 10^−5^) SNPs were 416 for RTs, 466 for PCs and 425 for CTs, each with a FDR < 0.01. These numbers are greater than the number of significant SNPs detected in any single trait analysis. Each of the three meta-analysis was an approximation to a full multi-trait analysis because they used summary statistics. However, they largely agreed with 368 SNPs being significant in all 3 meta-analyses (Fig. 3b, Supplementary Figure S2) and significant regions covering or close to previously reported loci in the dairy populations (Fig. 3 and Supplementary Figure S2). These loci included *DGAT1* (chromosome, chr14, 1.8 M+)^28^, *SDC2* (chr14, 69 M+)^15^, *GHR* (chr20, 31 M+)^15^, *CSN2* (chr6, 87 M+)^15^, *MGST1* (chr5, 93 M+)^15, 25^, *PAEP* (chr11, 103 M+)^30^ and *GPAT4* (chr27, 36 M+)^29^. The significance peak on chr18, 57 M+, detected in our study overlapped with reported *CTU1* locus associated with calving difficulty^27^. The small peak detected on chr3 (15.6 M+) was associated with previously identified *MUC1* locus^26^.

**Figure 4:**
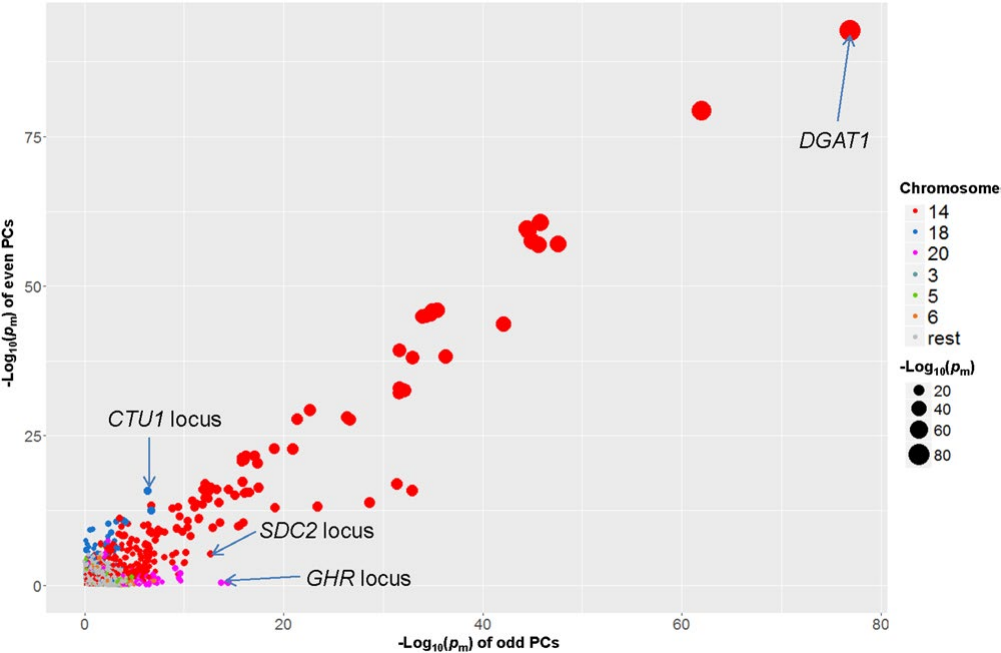
Relationship of multi-trait meta-analysis significance (*p*_m_) between odd (e.g., 1, 3, 5…) and even (2, 4, 6…) principle components (PCs). Some known loci affecting milk traits were highlighted.

Meta-analysis based only on the odd (e.g. 1, 3, 5…) or only on the even (2, 4, 6…) PCs again detected pleiotropic QTL on chromosome 14 and 18 (Fig. 4). SNPs on chromosome 14, especially around the DGAT1 locus, had a highly significant effect on both the even and odd PCs, confirming strong pleiotropic effects (Fig. 4). Other SNPs from loci of *SDC2* and *CTU1* were also significant in both odd and even PCs but not as consistently as DGAT (Fig. 4).

### Lead SNPs for dairy cattle

21 lead SNPs were selected based on the most significant SNP(s) from each chromosome of the dairy bulls (Supplementary Table S5) and were tested for their effects in the dairy cows. All 21 had an effect in the cows in the same direction as in the bulls and for >=17 SNPs this effect was significant (*p*_*v*_ < 0.05, Table 2).

**Table 2:** Summary of validation for raw traits (RTs), principal components (PCs) and Cholesky transformed traits (CTs).

SNP selection	Phenotype	SNPs no.	SNP no. with consistent effect directions^1^	Percent	SNPs no. *P* < 0.05 in validation GWAS^2^	Percent
Dairy cattle lead	RT	21	21	100%	17	81%
	PC		21	100%	18	86%
	CT		21	100%	17	81%
Dairy and beef cattle overlapped	RT	14	14	100%	4	29%
	PC		12	86%	4	33%
	CT		14	100%	5	36%

^1^The SNP effects are generated by genome-wide association studies (GWAS) using linear index as phenotype with cow data (validation population) and compared with the effect directions with GWAS of bulls (discovery population). ^2^The significance was determined by GWAS using linear index as phenotype with cows (validation population).

**Figure 5:**
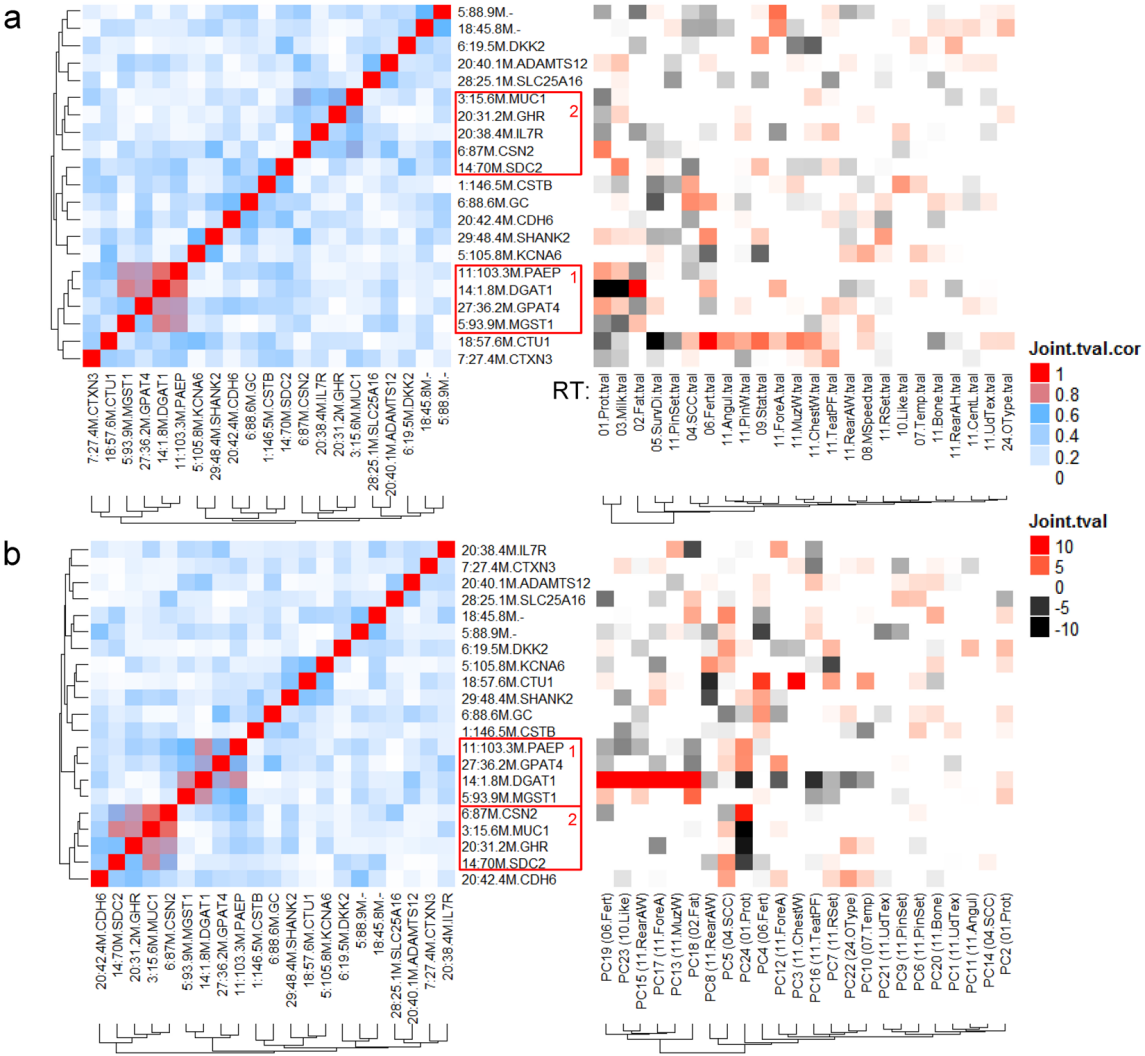
Clustering of lead SNPs representing major loci affecting dairy raw traits (RTs, **a**) and principal components (PCs, **b**) in the discovery population. Loci displaying similar effect clustering patterns across RT, PC and Choleskey transformed traits (Supplementary Figure S3) were highlighted in red boxes. Loci labels on the Y-axis were the same for both left (correlation of SNPs’ effects) and right (SNP’s effects on traits) panels. t values with absolute values >= 1 and validated for consistent effect directions between the discovery and validation populations were coloured. The RT associated with the top factor loading value of each PC was shown in the parentheses on the right panel (Supplementary Table S4).

Two clusters of lead SNPs were identified which had somewhat similar patterns of effects across traits, especially protein, milk and fat yield for RT, PC (Fig. 5a,b) and CTs (Supplementary Figure S3). The 1^st^ cluster included *DGAT1* (chr14, 1.8 M+), *MGST1* (chr5, 93 M+), *PAEP* (chr11, 103 M+) and *GPAT4* (chr27, 36 M+) loci. These SNPs had an allele which increased fat yield but decreased protein and especially milk yield (Fig. 5a). This effect pattern was consistent for the SNP effects on CT protein, milk and fat yield (Supplementary Figure S3). The 1^st^ cluster members also had correlated effects on PC18 and PC19 with top factor loading values associated with fat yield and fertility, respectively (Supplementary Table S4, Fig. 5b). The 2^nd^ cluster included *CSN2* (chr6, 87 M+), *MUC1* (chr3,15.6 M+), *GHR* (chr20, 29 M+) and *SDC2* (chr14, 69 M+), the clustering was stronger across PCs (Fig. 5b) and CTs (Supplementary Figure S3). These SNPs had an allele that increased CT protein but decreased CT milk (Supplementary Figure S3).

### Shared pleiotropic SNPs between dairy and beef cattle

**Figure 6:**
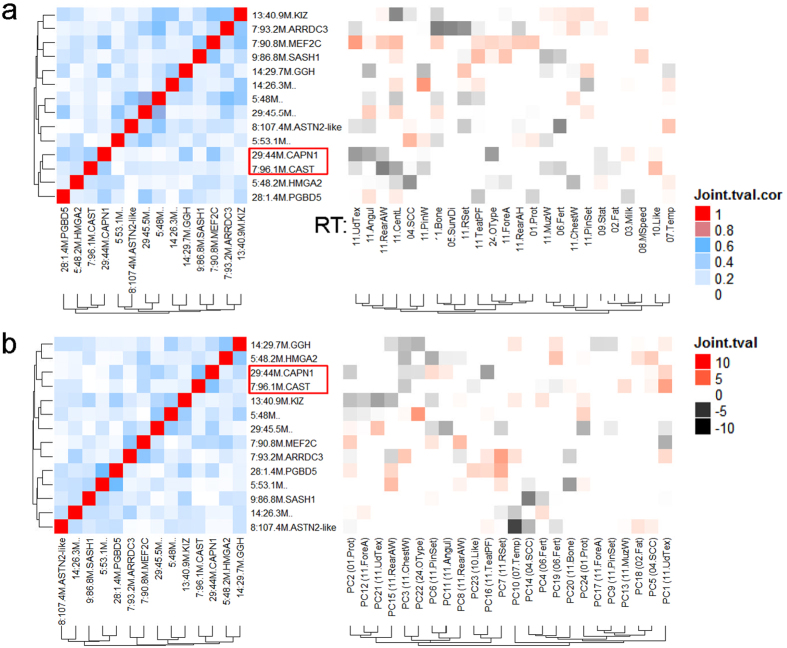
Clustering of the dairy-beef overlapped SNPs on raw traits (RTs, **a**) and principle components (PCs, **b**) of the dairy discovery population. Loci displaying similar effect clustering patterns across RT, PC and Choleskey transformed traits (Supplementary Figure S4) were highlighted in red boxes. Loci labels on the Y-axis were the same for both left (correlation of SNPs’ effects) and right (SNP’s effects on traits) panels. t values with absolute values > = 1 and validated for consistent effect directions between the discovery and validation populations were coloured. The RT associated with the top factor loading value of each PC was shown in the parentheses on the right panel (Supplementary Table S4).

Significant overlaps (*P*_f_ < 0.05) of pleiotropic SNPs were detected between the 25 dairy cattle traits and 15 beef cattle traits which included body height, muscle and fat mass. Although these dairy-beef shared SNPs had small effects in the dairy cattle population, more than 86% had effects in the same direction in bulls and cows (Table 2 and Supplementary Table S5). Many multi-trait identified dairy-beef shared SNPs had consistent effects for each single RT, PC and CT in the dairy discovery (bulls) and validation (cows) populations, although a majority of these effects were on body type traits rather than milk related traits (Fig. 6, Supplementary Figure S4 and Supplementary Table S7). We found that two SNPs related to *CAPN1* (29, 43 M+) and *CAST* (7, 96.1 M+), both associated with beef tenderness^7, 31^ showed correlated effect patterns across dairy cattle RTs, PCs and CTs. Both of these two loci contained an allele which decreased RT udder texture (11.UdTex), rear attachment width (11.RearAw) and stature, but increased likeability (farmer’s preferences score) (Fig. 6a). The likeability was the only trait with consistent effects of these two SNPs on CTs (Supplementary Figure S4). In addition, the *CAPN1* allele increased both beef tenderness and dairy milk yield, whereas the *CAST* allele increased beef tenderness but decreased dairy fat yield.

## Discussion

### Genetic properties of raw traits (RT), principal components (PC) and Cholesky transformed traits (CT)

Comparable with the previous results where daughter trait deviations of the dairy bulls were also analysed^9^, we identified the mid-to-high range of heritability estimates of bull’s RTs (Table 1). Using the trait deviation of multiple progenies as the sire’s phenotype reduced the amount of errors in the bull’s phenotypic data, thus, led to high estimation of heritability.

Single-trait GWAS of 25 correlated RT found many SNPs that were associated with more than one trait. This is not unexpected when the traits are correlated. However, after transforming the RT to uncorrelated PC and CTs, there were still many pairs of uncorrelated traits that shared significant SNPs. Cholesky decomposition has been widely used in twin studies to understand genetic covariances between traits^32^. However, our study appears to be the first one to use many CTs as uncorrelated traits for GWAS and detection of pleiotropy. Our results show great utility of CT in GWAS and detection of pleiotropy, especially better interpretability than PC as each CT is linked to at least one RTs. Another possible advantage of CT is that they can be calculated even when not all individuals have all traits, provided the traits can be ordered so that individuals with trait *k* recorded also have traits 1 to k-1 recorded. The current order of the RTs used to calculate CTs in our study was not the only possible choice as all studied cattle had complete phenotypic records. However, since the *k*th CT can be interpreted as the *k*th RT corrected for the preceding *k*-1 RTs, a biologically sensible order of RTs may improve the interpretability of the results of CT. Such order may require some prior knowledge of studied traits.

The PC dimension reduction approach^8^ hypothesized that genes act through a limited number of physiological pathways to impact on phenotypic traits. However, our results do not support this hypothesis as all PCs appear to be genetically important (Table 1). This is consistent with the previous report in human where genetic information of all PCs were used to achieve maximum GWAS power^10^. If the genes in a pathway had a similar pattern of effects across traits, this pattern would emerge as a PC and the overall correlations between traits would reflect this pattern. In this case, SNPs would be associated with only one PC, many SNPs would show the same pattern of effects across traits and this pattern would be in line with the overall correlations. None of these predictions were confirmed by our results. This is exemplified by SNPs within *DGAT1* which have significant effects on several PCs (Fig. 5b). We further confirmed this by meta-analysis of single-trait GWAS of odd PCs and single-trait GWAS of even PCs where *DGAT1* showed the most consistent significances (Fig. 4). The spread effects of *DGAT1* on PCs occurs because the effects of *DGAT1* do not follow the pattern described by the overall genetic correlations. For instance, RT milk and fat yield are positively correlated but the allele of *DGAT1* that increases fat yield decreases milk yield (Fig. 5a). These findings imply that causal variants act through diverse rather than a limited number of biological pathways to affect different traits. This conclusion is supported by the cluster analysis of lead SNPs. Although 2 clusters were identified, the SNPs within a cluster only partially share the same pattern of effects across traits. This limited sharing is possibly explained by competition for substrates between different synthesis pathways within the mammary gland (see below).

We show that using genetic information of all PCs can be more powerful than using a limited number PCs in understanding pleiotropy. However, the PC results themselves had limited interpretability at the phenotypic level (Supplementary Table S2). Previously, factor analysis^33^ showed ability to distinguish latent pathways in dairy cattle phenotypes^34^ but this was based on closely related traits describing fatty acid profiles. A future analysis with interpretable latent factors may improve our understanding of biology of the animal.

### Pleiotropy in the dairy cattle

Our findings show that there is a substantial amount of pleiotropy detectable in correlated RT and uncorrelated PC and CT at both single-trait and multi-trait level (Figs 1–3). The three meta-analyses, although each is an approximation, yielded similar results. The powerful multi-trait approach (P < 1 × 10^−5^ and FDR < 0.01) identified many significant pleiotropy SNPs with 100% validation rates of 21 lead SNPs (Table 2). These results demonstrate the existence of major pleiotropic loci in the dairy cattle population affecting uncorrelated traits, which are independent of spurious pleiotropy, i.e., cross-phenotype^4^.

The lead SNPs representing the dairy cattle major pleiotropic loci in Holstein, Jersey and Australian Red, largely overlap with reported dairy production-traits related loci (Fig. 5). Using cluster analysis RTs, PCs and CTs, we identified two clusters of SNPs. Within a cluster the SNPs had a similar pattern of effects across traits but this was largely restricted to milk production traits. Overall the evidence for clustering of the effects of loci was weak, suggesting that each locus had a unique pattern of effects across traits. The 1^st^ cluster contained SNPs close to the loci *DGAT1* (chr14, 1.8 M+)^9, 15, 26^, *MGST1* (chr5, 93 M+)^25^, *PAEP* (chr11, 103 M+)^26^ and *GPAT4* (chr27, 36 M+)^29^, each of which has an allele that increases fat yield but decreases protein and milk yields. *DGAT1*, *MGST1* and *GPAT4* have an effect on fat synthesis but *PAEP* is the gene for the milk protein beta lactoglobulin. The simplest explanation for this clustering is competition for substrate within the mammary gland. That is, a mutation in *DGAT1* that decreases fat synthesis causes more substrate to be available for lactose and protein synthesis. In human cell lines, *DGAT1* and *MGST1* were reported to show co-expression in a gene group regulating adipogenesis^35^.

The 2^nd^ cluster contained *CSN2* (chr6, 87 M+), *MUC1* (chr3,15.6 M+), *GHR* (chr20, 31.2 M+) and *SDC2* (chr14, 69 M+). Each member has an allele that increases protein yield but decreases milk yield (Fig. 5 and Supplementary Figure S3). Again this might be the result of competition for substrates within the mammary gland. Individually, *MUC1* ^36^, *GHR* ^15, 26^, *CSN2* ^15^ and *SDC2* ^15^ loci were reported for their major effects on protein and/or milk yield, but not on fat yield. In our study also, these four loci showed weak or no effects on fat yield. It is likely that the 2^nd^ cluster SNP members contribute to milk production variations differently from the mechanisms allowing the 1^st^ cluster to impact on milk production. However, to clarify the exact physiological differences between these two clusters in affecting milk production, more precisely measured phenotype will be required. Nevertheless, both the cluster analysis and above PC analysis lead to the conclusion that the genes affecting these 25 traits do not work through a small number of pathways. Rather almost every gene seems to have its own pattern of effects across traits.

Pleiotropy analysis can also extend our knowledge of known SNP for their unknown effects. Previously reported association between *CTU1* and calving difficulty in the Holstein-Friesian cattle^27^ was consistent with our observation of strong *CTU1* effects on RT, PC and CT fertility (Fig. 5 and Supplementary Figure S3). However, we also identified *CTU1* as one of the strongest pleiotropic loci (Figs 3 and 4) with a wide range of effects on non-production dairy RTs and CTs, including decreasing fertility rate but increasing stature and chest width. Although the gene function of *CTU1* is unknown^27, 37^, its widespread effects on both production and non-production traits suggest that it is an important marker for a balanced selection of dairy cattle.

### Shared pleiotropic SNPs between dairy and beef cattle

Some SNPs with large effects on beef cattle traits were shown to have small effects on dairy traits. (Fig. 6). The two SNPs with the most consistent effect patterns across RTs, PCs and CTs were close to *CAPN1* (29, 43 M+) and *CAST* (7, 96.1 M+). Previously, these two SNPs were reported for both independent^7^ and epistatic^31^ effects on beef tenderness and the CAST protein binds to CAPN1 to inhibit its activities^38^. The effects on tenderness are partially post-mortem but in live cattle these proteins probably affect protein turnover. However, we did not observe significant effects of these two loci on protein yield in dairy cattle. Instead, the majority of observed effects of these two SNPs were on conformation traits (Fig. 6 and Supplementary Figure S4) which may eventually lead to their consistent SNP effects on likeability. *MEF2C* was identified as one of the most active transcription factor along with *Myostatin* to regulate muscle growth in the beef cattle^39^ and had functional variants related to growth in ruminants^40, 41^. The observed effects of the SNP close to *MEF2C* (0.07 Mb away) on a wide range of body type traits appear to be consistent with its role in regulating muscle development and growth.

We identified less characterised loci, including SNPs within gene *KIZ* (13, 40 M+) and within *PGBD5* (around 28, 1.4 M) and close to *SASH1* (9, 86+) with widespread pleiotropic effects on dairy RTs, PCs and CTs (Fig. 6a and Supplementary Figure S4). Consistent with a recent report from Canadian Holstein cattle, SNPs within *KIZ* (*PLK1S1*) had effects on somatic cell count but not on milk production traits^42^. However, we also identified its significant effects on milking speed (in both RT and CT). RT and CT milking speed are also associated with the SNP close to *SASH1* which regulates cell proliferation and apoptosis in human (RefSeq). There is too limited knowledge of the function of the piggyBac transposable element derived gene *PGBD5* for us to explain its pleiotropic effects, especially these effects on RT, PC and CT protein yield, in the dairy cattle. Interestingly, PGBD5 gene is highly conserved across invertebrates and its encoded protein affected many other genes’ actions by inducing DNA transposition and may contribute to complex traits by genome remodelling^43^. It may also have an important regulatory role in the ruminant species.

We conclude that SNPs with a large effect on one trait are likely to have much smaller effects on other traits. Transforming the correlated traits to uncorrelated PCs or CTs reduces but does not eliminate this pleiotropy at the single-trait GWAS level. We hypothesise that this is because each causal variant has a unique pattern of effects across traits and hence generates a set of correlated effects that is different to the average correlations caused by all the causal variants together. Consequently, when the average correlations are used to construct PCs or CTs, some causal variants still have effects on multiple uncorrelated traits. A previously less characterised locus CTU1 (chromosome 18, 57 M+) showed strong pleiotropic effects on production and non-production traits. We also identified SNPs with strong effects in beef cattle showing small but validated effects in dairy populations. Our findings are not only useful for the dairy industry to use multi-functional genetic markers to achieve efficient selection, also provide important information for other researchers to consider when conducting multi-trait genome-wide analysis.

## Electronic supplementary material

Supplementary Table

Supplementary Figure
