# Supplementary material for: Genome-wide comparative analyses of correlated and uncorrelated phenotypes identify major pleiotropic variants in dairy cattle
Source: Sci Rep. 2017 Aug 23;7:9248. doi: 10.1038/s41598-017-09788-9 (PMC5569018; doi:10.1038/s41598-017-09788-9)
Supplement: Supplementary file 2 — Supplementary Figure [file 41598_2017_9788_MOESM2_ESM.pdf]

Title page:

Supplementary Figure S1-4 for manuscript:

Genome-wide comparative analyses of correlated and uncorrelated phenotypes identify major pleiotropic variants in dairy cattle

Ruidong Xiang[1,2,*] Iona M. MacLeod[2], Sunduimijid Bolormaa[2,3] and Michael E. Goddard[1,2]

[1]Faculty of Veterinary & Agricultural Science, University of Melbourne, Victoria 3010, Australia.
[2]AgriBio, Dept. Economic Development, Jobs, Transport & Resources, Victoria, Australia.
[3]Cooperative Research Centre for Sheep Industry Innovation, Armidale, NSW 2351, Australia.

*Corresponding author: Ruidong Xiang (ruidong.xiang@unimelb.edu.au)

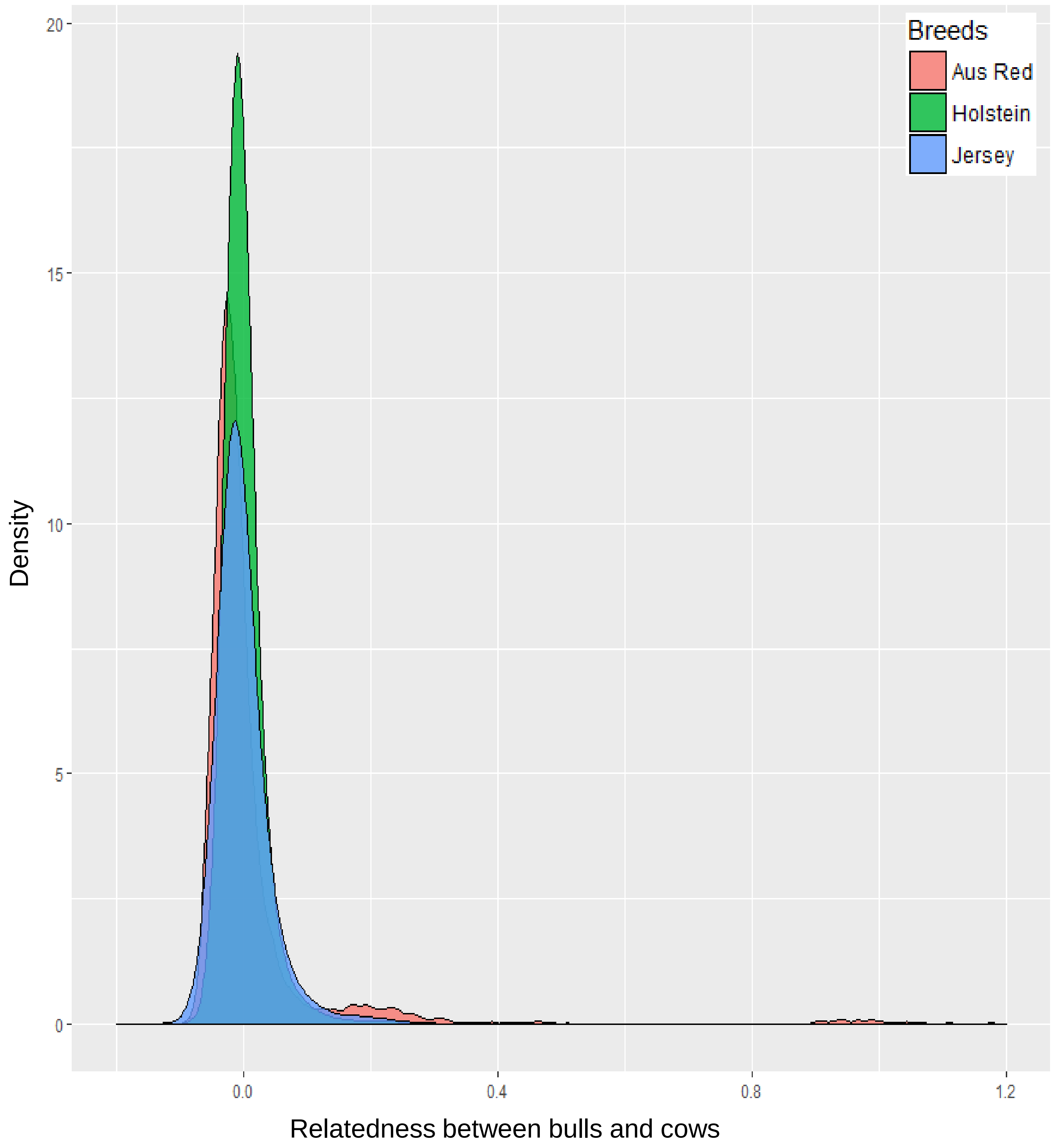

Figure S1: Density plot of the genomic relationship matrix between bulls and cows.

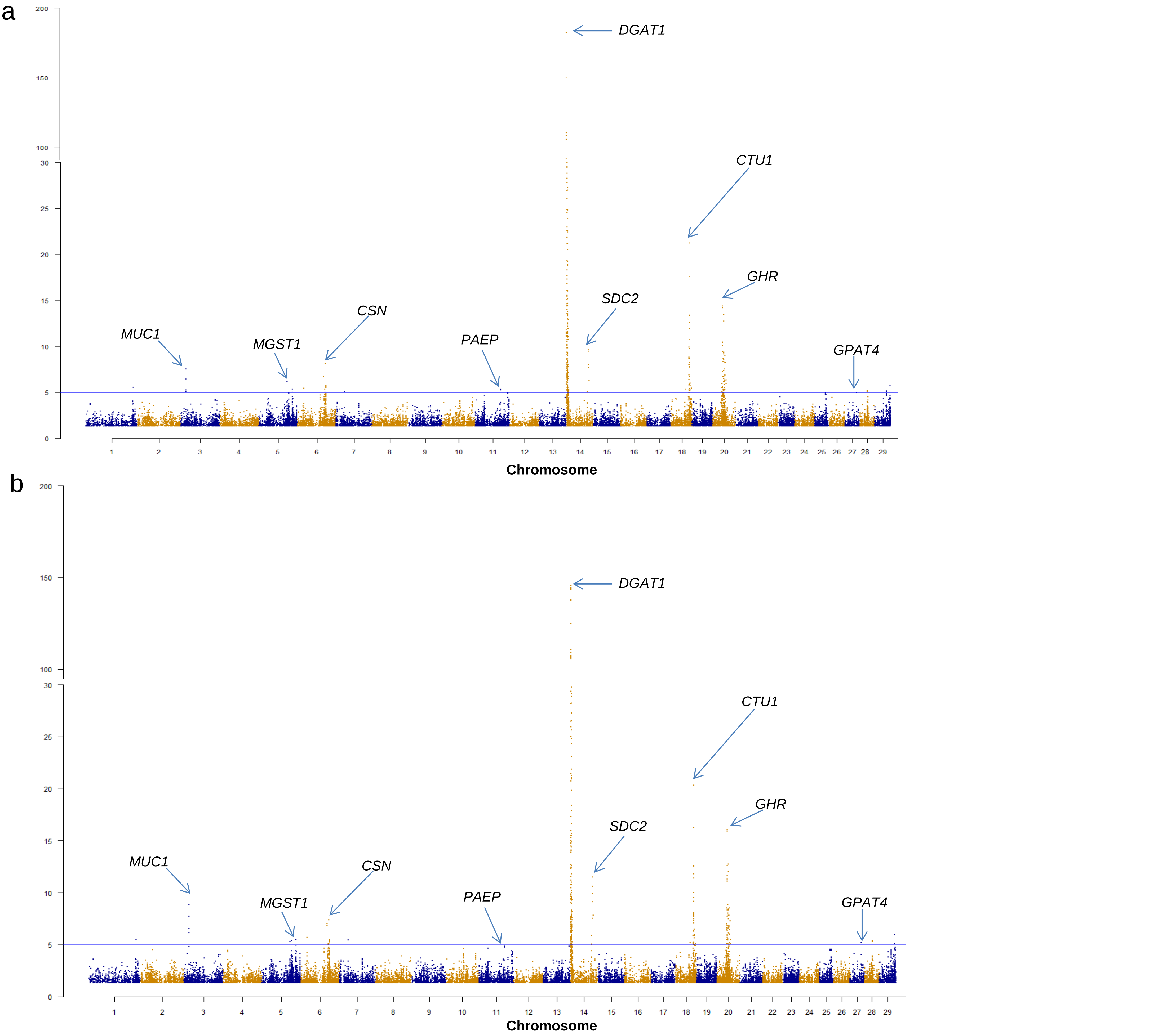

Figure S2. Manhattan plots of multi-trait meta-analysis for bull principal components (PCs, a) and Cholesky transformed traits (CTs, b). Only SNPs with P<0.05 were input. The horizontal blue line was the significance of 0.00001. Some reported loci affecting milk traits were highlighted.

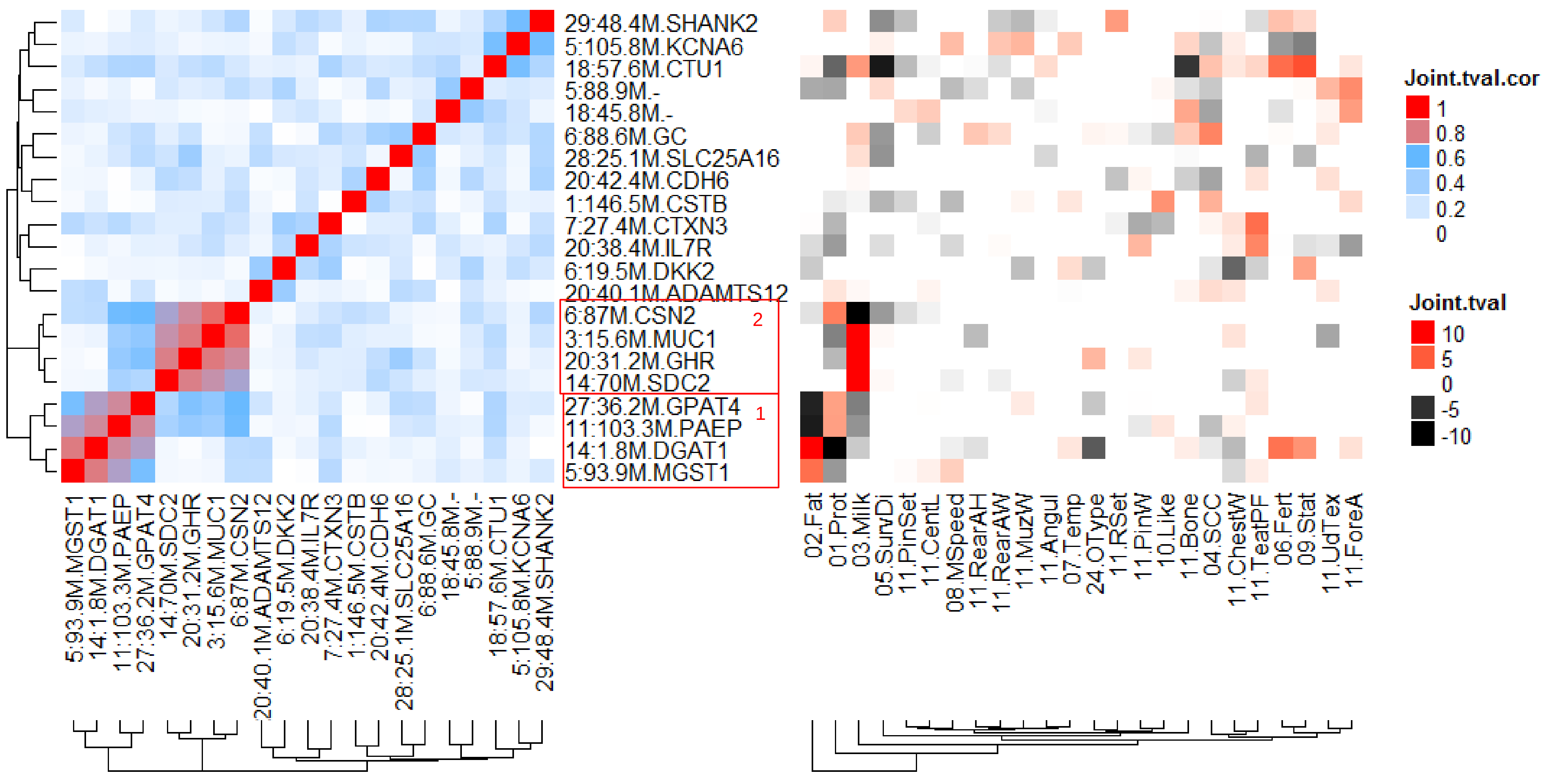

Figure S3. Selected lead SNPs representing major QTLs affecting Choleskey transformed traits (CTs) the discovery data. Loci displaying similar effect clustering patterns across raw traits, principle components (Figure 5) and CT were highlighted in red boxes. The clusters of correlations of t values (left heatmap) and directions for RTs (right heatmap) were based on 'joint' analysis (J. Yang et al 2012) which fitted all selected lead SNPs simultaneously in one regression per one CT. t values with absolute values >= 1 and validated for consistent effect directions between the discovery and validation populations were coloured for each CT.

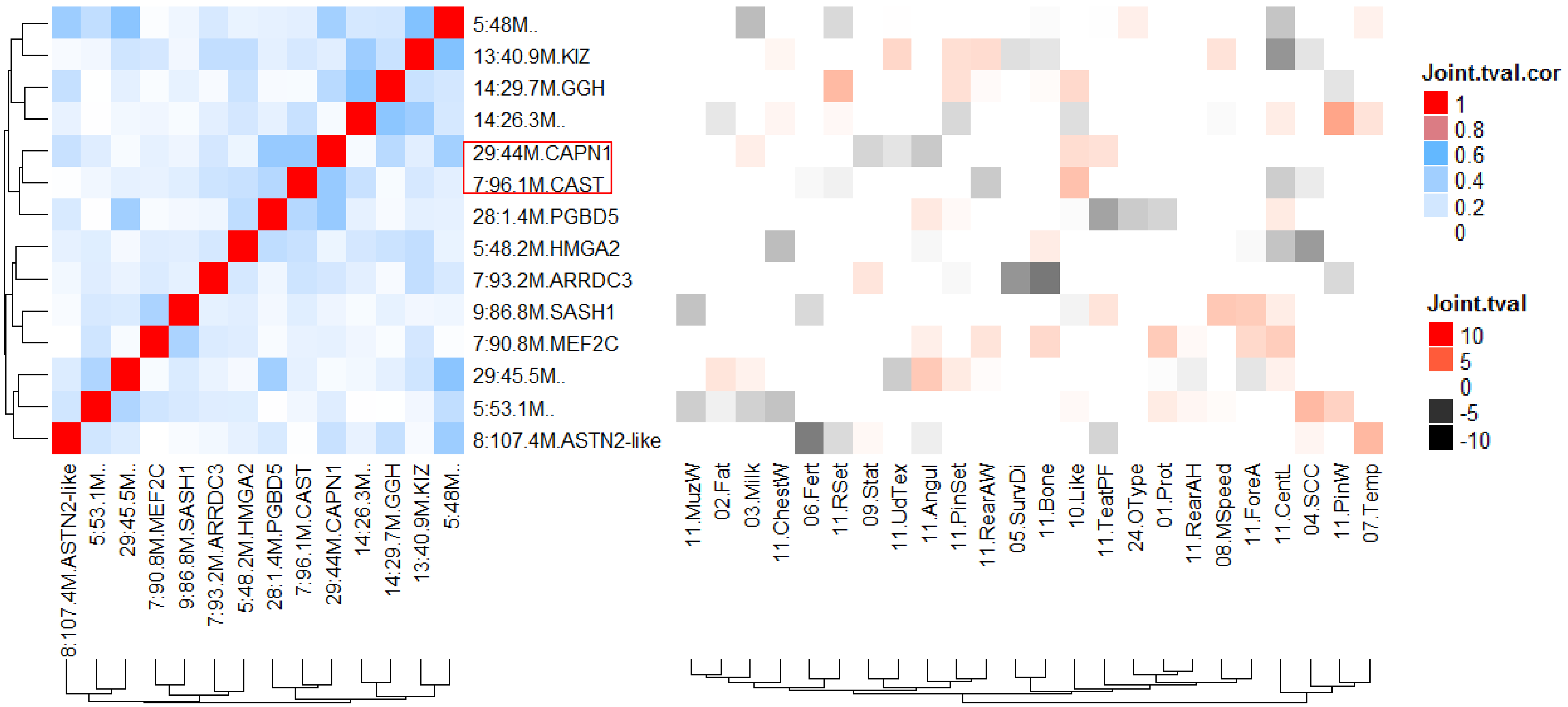

Figure S4. The effects of the dairy beef overlapped SNPs on Choleskey transformed traits (CTs) of the dairy discovery population. Loci displaying similar effect clustering patterns across raw traits, principle components (Figure 6) and CTs were highlighted in red boxes. The clusters of correlations of t values (left heatmap) and directions for RTs (right heatmap) were based on 'joint' analysis (J. Yang et al 2012) which fitted all selected lead SNPs simultaneously in one regression per one CT. t values with absolute values >= 1 and validated for consistent effect directions between the discovery and validation populations were coloured for each CT.
